# Effect of Hydrothermal Treatment on the Structure and Functional Properties of Quinoa Protein Isolate

**DOI:** 10.3390/foods11192954

**Published:** 2022-09-21

**Authors:** Xingfen He, Bin Wang, Baotang Zhao, Yuecheng Meng, Jie Chen, Fumin Yang

**Affiliations:** 1 School of Food Science and Biotechnology, Zhejiang Gongshang University, Hangzhou 310018, China; 2 College of Food Science and Engineering, Gansu Agricultural University, Lanzhou 730070, China

**Keywords:** quinoa (*Chenopodium quinoa* Willd), protein isolate, hydrothermal treatment, structure, functional properties

## Abstract

The aim of this study was to investigate the effects of hydrothermal treatment at different temperatures and times on the structure and functional properties of quinoa protein isolate (QPI). The structure of QPI was investigated by analyzing changes in the intrinsic fluorescence spectrum, ultra-violet (UV) spectrum, and Fourier transform infrared spectrum. The solubility, water/oil-holding capacity, emulsifying activity, and emulsion stability of QPI were studied, as were the particle size and the thermogravimetric properties of QPI. The results showed that the average particle size of QPI gradually increased with the increase in hydrothermal treatment time and temperature, and reached a maximum value of 121 °C for 30 min. The surface morphology also became rough and its thermal stability also increased. The endogenous fluorescence and UV spectral intensity at 280 nm decreased gradually with increasing hydrothermal treatment time and temperature, and reduced to the minimum values at 121 °C for 30 min, respectively. After hydrothermal treatment, the secondary structure of QPI tended to be disordered. The functional properties of QPI after treatment were all superior to those of the control. The results of this study might provide a basis for the processing and utilization of QPI.

## 1. Introduction

*Chenopodium quinoa* Willd is a kind of quasi-grain. Its protein content is 12.0–23.0%, which is higher than rice, maize and barley [1]. Quinoa protein isolate (QPI) is rich in all the essential amino acids needed by the human body, with a balanced amino acid content, and is easily absorbed by the human body [2]. Quinoa protein is mainly composed of 37% 11S globulin and 35% 2S albumin; disulfide bonds are the key to stabilizing the protein structure, while gluten and gliadin are less so [3]. In addition,11S globulin is a hexamer composed of a 22–23 kDa basic group and a 32–39 kDa acidic group [4]. 2S Albumin is a heterodimer linked by about 30–40 and 60–90 residues via disulfide bonds (Mw 8–9 kDa) [5]. Compared with most grain proteins, QPI is closer to milk and meat, and is a sustainable high-quality plant protein. In recent years. QPI has gradually become a research hotspot due to its good functional and physicochemical properties that could be used in the food industry [6]. Studies have shown that the emulsifying activity and emulsion stability of QPI are higher than those of wheat protein and soybean protein [7]. QPI has strong water and oil-holding capacities which are higher than those of oats, soybeans, and wheat proteins [8,9,10].

The structure and functional properties of protein determine its application scope in food processing. The commonly used modification methods in the food industry include ultrasound, high pressure, pH and heat treatment, among which heat treatment has attracted wide attention due to its simple operation and low cost [11,12,13,14,15]. Heat treatment causes thermal denaturation through the destruction of covalent bonds, which increases the exposure of hydrophobic and thiol (SH) groups, and has a substantial impact on the functional properties of food proteins such as solubility, foaming, and emulsifying properties [11]. Heat treatment and alkali treatment can improve the performance of rice protein [16]. Ultrasonic heat treatment reduced the relative content of α-helix and β-sheet in soybean protein secondary structure, and increased the relative content of random coil, resulting in loose tertiary structure [17]. Heat treatment significantly affects the conformation of *Cuminum cyminum* protein, resulting in a surface hydrophobicity increase [15]. Heat treatment at 85 °C for 15 s increased the particle size, turbidity, zeta potential, and surface hydrophobicity of goat milk proteins, further improving their functional properties [18]. Studies also have shown that heat treatment has an effect on the structural and functional properties of faba bean protein [19], protein isolate from *Stauntonia brachyanthera* seeds [20], and sunflower protein [21]. Up to the present, there are few reports indicating the potential effect of heat treatment on QPI. Previous studies have shown that different kinds of heat treatments significantly affected the structural and functional properties of QPI [22]. In addition, the degree of aggregation of QPI can be changed by adjusting the hydrothermal treatment conditions [23]. The water retention capacity improved considerably in the heat-modified and frozen QPI [24]. Mir et al. (2021) have investigated the effects of heat treatment at 80, 90, and 100 °C for 15 and 30 min on the functional properties of QPI [25]. However, the aims of present study are to analyze the effects of the ordinary hydrothermal treatment and simulated autoclaving hydrothermal treatment on the new variety “Longli-1” quinoa protein isolate. We focused on the effects of ordinary hydrothermal treatment and simulated autoclaving on the secondary and tertiary structure of QPI and their effects on functional properties, which are important for processing. The results of this study provide some valuable information for the application of quinoa protein in a variety of foods.

The aims of this study were to (1) determine the changes in the particle size of quinoa protein under different temperature and time hydrothermal treatments; (2) investigate the effects of hydrothermal treatment conditions on the thermal stability of quinoa protein; (3) study how hydrothermal treatment affects the secondary and tertiary structure of quinoa protein; and (4) measure the different functional properties of quinoa protein after hydrothermal treatments.

## 2. Materials and Methods

### 2.1. Materials

The “Longli-1” quinoa was kindly supplied by Gansu Academy of Agricultural Sciences (Lanzhou, China). After mechanical shelling, quinoa was packed in woven bags and the samples were stored at room temperature (22 ± 3 °C, RH 55–60%) for 7 d. Sodium dodecyl sulfate (SDS) and phosphate buffer saline were purchased from Shanghai Yuanye Biological Technology Co., Ltd., (Shanghai, China). Edible soybean oil was purchased from a local supermarket.

### 2.2. Isolation of QPI

The quinoa seeds were crushed and passed through a 60-mesh sieve. The quinoa powder was defatted (petroleum ether, 30–60 °C) 3 times and placed in a fume hood to air dry. The defatted quinoa flour and deionized water were mixed in a ratio of 1:20 g·mL^−1^ and the suspension was then prepared. This suspension was adjusted to pH 10, stirred for 2 h in a water bath at 47 °C, and centrifugated (5000 rpm, 15 min) to take the supernatant. The pH of the supernatant was adjusted to 4.5 (1 M HCl) and the precipitate was collected by centrifugation (5000 rpm, 15 min, 4 °C). The precipitate was reconstituted and washed with deionized water 5 times. The washed precipitate was redissolved in phosphate buffer saline solution at pH 7.0 and placed in a dialysis bag with a molecular retention of 8 kDa. Then the dialysis bag was placed in primary water for dialysis for 48 h (4 °C), and the water was replaced every 3 h. The suspension was vacuum freeze-dried (LyoQuest-85, Telstar Lab, Madrid, Spain) to obtain QPI [26].

### 2.3. Hydrothermal Treatment of QPI

A 5% QPI solution was prepared using phosphate buffer saline and treated at different temperatures of 25, 60, 70, 80, 90, 100, and 121 °C for 5, 10, 20, and 30 min (60–100 °C was carried out in a water bath, HH-4, Guohua Electric Co., Ltd., Shanghai, China, 121 °C was carried out in an autoclave, MLS-3750, SANYO Corporation, Osaka, Japan), and then quickly cooled. (25 °C in the text refers to QPI without hydrothermal treatment, i.e., natural QPI, which is recorded as control.) The hydrothermally treated QPI solution was vacuum freeze-dried (LyoQuest-85, Telstar Lab, Madrid, Spain) to obtain freeze-dried QPI [27].

### 2.4. Structural Properties of QPI

#### 2.4.1. Particle Size 

Laser particle size analyzer (Bettersize 2600, Dandong Better Instruments Co., Ltd., Dandong, China) was used to measure the particle size of the QPI. The measurement temperature was 25 °C, the refractive index of the sample and the dispersant were 1.46 and 1.33, respectively, and the refractive index was 1.20–1.40% when measured. The QPI was added to cuvette dropwise till the refractive index reached between 5.00% and 10.0% [28].

#### 2.4.2. Thermogravimetric Characteristics

The thermogravimetric analysis of QPI was determined by TGA (TGA 550, TA Instruments Co., Ltd., New Castle, DE, USA). Freeze-dried QPI (5–10 mg) was placed in a platinum–rhodium alloy tray, the scan rate was set to 50 °C/min, and the temperature range was 50 to 700 °C. TGA was performed on QPI under nitrogen atmosphere [29].

#### 2.4.3. Intrinsic Fluorescence Spectrum

Intrinsic fluorescence of QPI was determined using a Fluorescence spectrophotometer (F-4700, Hitachi High-Tech Science Co., Ltd. Naka Office, Naka, Japan). Freeze-dried QPI was mixed with phosphate buffer saline to prepare 0.15 mg/mL QPI solutions (the solution was filtered through a 0.45 μm aqueous filter). The setting parameters were: excitation wavelength 290 nm, emission spectrum was recorded in the range of 300–460 nm, and excitation and emission slits were both 5 nm [30].

#### 2.4.4. Ultra-Violet (UV) Spectrum

Freeze-dried QPI was mixed with phosphate buffer saline to prepare 1 mg/mL QPI solutions (the solution was filtered through a 0.45 μm aqueous filter), and performed UV spectrum scanning (UV-2450, Shimadzu Instruments Co., Ltd., Tokyo, Japan). The scanning wavelength and scanning rate were set to 200–400 nm and 2 nm/s, respectively [31].

#### 2.4.5. FTIR

FTIR of freeze-dried QPI was characterized using an FTIR spectrum (FTIR920, Tianjin Tuopu Instrument Co., Ltd., Tianjin, China). The freeze-dried QPI and KBr were mixed uniformly at a ratio of 1:200, then pulverized and compressed for determination. The measurement temperature was the ambient temperature (25 °C), the wave number, resolution and wave number accuracy were set to 400–4000 cm^−1^, 4 cm^−1^ and 0.01 cm, respectively, and the number of scans was 64 times [32].

### 2.5. Determination of Functional Properties of QPI

#### 2.5.1. Solubility

The freeze-dried QPI was dissolved in phosphate buffer saline pH 7 (0.500%), magnetically stirred at ambient temperature (25 °C) for 20 min, and then centrifuged (4000 rpm, 20 min) [33].

The solubility of QPI was expressed as follows:

(1) $$\mathrm{Solubility}/\;\%=\frac{\mathrm{Protein}\;\mathrm{content}\;\mathrm{in}\;\mathrm{supernatant}}{\mathrm{Total}\;\mathrm{protein}\;\mathrm{content}\;\mathrm{in}\;\mathrm{the}\;\mathrm{sample}}\times 100$$

The protein content in the supernatant, i.e., the content of soluble protein, was determined by the Coomassie brilliant blue method (CCB) [34]. The specific steps were as follows: Coomassie brilliant blue G-250 was used for color development, and bovine serum albumin (BSA) was used as the reference substance. The content of soluble protein was determined by visible spectrophotometry at the detection wavelength of 595 nm (2700, UV–Vis Spectrophotometer, Co., Ltd. Shimadzu, Tokyo, Japan). The standard curve used was Y = 0.0009X + 0.1834 (R^2^ = 0.9898). The total protein content was determined by Kjeldahl method (total nitrogen × 6.38). In this method, 0.5 g freeze-dried quinoa protein powder was used as a sample for determination.

#### 2.5.2. Water-Holding Capacity (WHC) and Oil-Holding Capacity (OHC)

Freeze-dried QPI (0.5 g) was placed in a dry centrifuge tube and mixed with 10 mL of deionized water. The mixture was magnetically stirred at 25 °C for 30 min and then centrifuged (4000 rpm, 30 min). After decanting the supernatant, the centrifuge tube was tilted (45°) for 30 min to remove excess water. The total mass of the centrifuge tube and sediment was recorded. The water-holding capacity (WHC) was calculated using the equation:

(2) $$\mathrm{WHC}/\;\%=\frac{m_{2}-m_{1}}{0.5}\times 100$$

where m_1_ is the mass of freeze-dried QPI and centrifuge tube (g), m_2_ is the mass of sediment and centrifuge tube (g).

Freeze-dried QPI (0.5 g) was placed in a dry centrifuge tube and mixed with 3 mL of soybean oil. The mixture was vortexed at room temperature (25 °C) for 30 min and then centrifuged (4000 rpm, 30 min). After decanting the supernatant, the centrifuge tube was tilted (45°) for 30 min to remove excess oil. The mass of the sediment is recorded. The oil-holding capacity (OHC) was calculated as follows:

(3) $$\mathrm{OHC}/\;\%=\frac{m_{2}-m_{1}}{0.5}\times 100$$

where m_1_ is the mass of QPI (g), and m_2_ is QPI’s sediment (g) [35].

#### 2.5.3. Emulsifying Activity (EA) and Emulsion Stability (ES)

A mixed solution of 24 mL hydrothermal treatment QPI solution (1.00%, *w*/*v*) and 8 mL soybean oil was whipped with a high-speed shearing dispersing emulsifier (FA25, FLUKO Equipment Shanghai Co., Ltd., Shanghai, China) at 10,000 rpm for 5 min at 25 °C. The emulsion (0.05 mL) and 5 mL of sodium dodecyl sulfate (SDS) solution (0.100%) were mixed and immediately shaken to mix. The absorbance of the emulsion after being placed for 0 min and 10 min were measured at a wavelength of 500 nm with 0.100% SDS solution as a control. The emulsifying activity (EA) was calculated using the equation:

(4) $$\mathrm{EA}\;\left(m^{2}/g\right)=\frac{2\times 2.303\times A_{0}\times \mathrm{DF}}{C\times \rho \times \theta \times 10000}$$

where A_0_, DF, ρ, and θ are the absorbance value of the sample, dilution factor (100), optical path (1 cm), and oil volume fraction (0.25), respectively.

The emulsion stability (ES) was calculated as follows:

(5) $$\mathrm{ES}/\;\%=\frac{{\mathrm{EA}}_{10}}{\mathrm{EA}}\times 100$$

where EA_10_ is the emulsifying activity (m^2^/g) at 10 min after being placed [36].

### 2.6. Data Analysis

The indicators involved in the test were measured three times. All data were calculated using Excel 2007 (Microsoft, Redmond, WA, USA) to calculate the mean and standard deviation, Origin 8.5 (Origin Lab, Northampton, MA, USA) was used for graphing, SPSS 17.0 (International Business Machines, Armonk, NY, USA) was used for one-way ANOVA, and Peak Fit V4.12 (Reachsoft, Beijing, China) was used for fitting analysis of infrared spectra.

## 3. Results and Discussion

### 3.1. Structural Properties of QPI

#### 3.1.1. Particle Size

**Figure 1:**
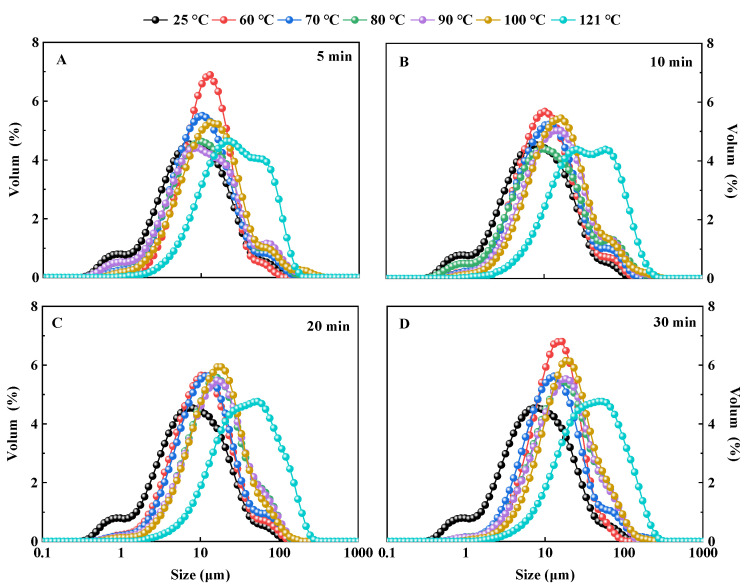
Effects of hydrothermal treatment of 5 min (**A**), 10 min (**B**), 20 min (**C**), 30 min (**D**) on particle size distribution of QPI.

The functional properties of proteins are affected by protein particle size [37]. The results showed that compared with the control, the particle size distribution of QPI after hydrothermal treatment became wider, and the overall distribution shifted to the right with the increase in hydrothermal treatment time and temperature, and 121 °C had the most significant effect on it (Figure 1A–D). The volumetric mean particle size D_[4,3]_ of QPI gradually increased with increasing hydrothermal treatment time and temperature, and the effect was most significant of 121 °C, reaching a maximum at 121 °C for 30 min, which was 3.31 times higher than the control (*p* < 0.05) (Table 1). D_[5,0]_ can reflect to some extent the aggregation of proteins, a key factor in the evaluation of protein quality [36]. D_[5,0]_ of QPI increased with increasing hydrothermal treatment time and temperature, reaching a maximum at hydrothermal treatment conditions of 121 °C for 30 min, which was significantly higher than the control by 3.93 times (*p* < 0.05) (Table 1).

We also found that the average particle size of QPI increased with the increase in temperature, and the degree of particle size inhomogeneity also increased, which is similar to the results from the study of the influence of heating on the particle size of lotus (*Nelumbo nucifera* Gaertn.) seed protein. The results showed that all heat treatments resulted in a significant increase in protein particle size compared to native QPI [38]. Previous studies showed that the particle size of rice gluten increased gradually during heat treatment [39]. The possible reasons may be due to hydrothermal treatment, which causes the 7S and 11S globulins in the protein to cross-link through disulfide bonds to form aggregates and the intact 11S globulin monomers to readily form covalent aggregates, leading to an increase in the particle size of the protein [40]. It is suggested that the hydrothermal treatment causes changes such as cross-linking or aggregation between protein molecules, generating a large number of aggregates, and the degree of protein aggregation increases during the heat treatment, which is consistent with the findings of Wang et al., (2020) [22]. In addition, similar results were found in a study on the effect of heat treatment on the particle size of sunflower protein isolates [21]. This suggests that hydrothermal treatment causes QPI to form aggregates leading to a significant increase in its particle size. However, the results are in contradiction with the results of Mir et al., (2021) who observed a reverse kind of trend whereby the particle size of all QPI samples after heat treatment was smaller than that of native QPI [25]. Among all the heat-treated QPI, the decrease in QPI particle size was the highest at 80 °C for 30 min, and the decrease in QPI particle size was the lowest at 100 °C for 30 min. However, in our results hydrothermal treatment significantly increased the particle size of QPI. The possible reasons for this may be due to the quinoa used in this study is a new variety, Longli-1, which is cultivated in China. Studies have shown that the chemical composition and amino acid profile of different varieties of grains are different, which has a great impact on the physicochemical properties of proteins, therefore affecting the degree of aggregation and particle size [41,42].

#### 3.1.2. Thermogravimetric Characteristics

**Figure 2:**
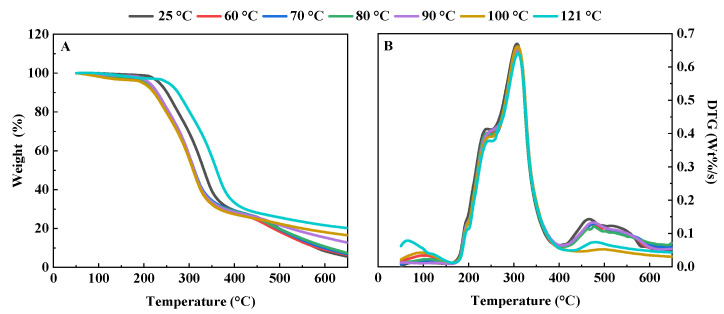
Effects of different hydrothermal treatment temperatures on thermogravimetric (**A**) and derivative thermogravimetric curve (**B**) of QPI.

The effect of hydrothermal treatment on the thermal stability of QPI was investigated by thermogravimetric analysis of QPI. The results showed that different temperatures had significant effects on the thermogravimetric properties of QPI. The thermal degradation of QPI was divided into three stages, the first stage was from 50 to 200 °C; the weight loss in this stage was due to the evaporation of residual water and the degradation of low molecular weight volatiles. The second stage was from 200 to 400 °C. As the temperature increased further, both non-covalent and covalent bonds in QPI broke, including covalent peptide bonds, disulfide bonds, O-O and O-N, resulting in the complete breakdown of the QPI protein backbone and the release of various gases, such as CO, CO_2_, and NH_3_ [43]. The third stage was from 400 to 700 °C, during which the slope of the TGA curve changed, the weight loss slowed down, and the degradation of the control began at about 200 °C, while the degradation of the QPI hydrothermally treated at 121 °C began at about 230 °C. This showed that the QPI after heat treatment had higher thermal stability, and when the heat treatment temperature was 121 °C, the thermal stability of QPI was the highest (Figure 2A). In all three degradation stages, the weight loss of QPI was lower after hydrothermal treatment compared to the control. A similar phenomenon was also found in phosphate-modified peanut protein isolates [44] and protein concentrate of an edible seaweed named *Kappaphycus alvarezii* (Doty) Doty [45]. Similar trends were observed when Malik and Saini investigated the thermogravimetric properties of heat-treated sunflower protein [46].

The derivative thermogravimetric (DTG) curve of QPI showed a unimodal change with a distinct peak (Figure 2B). Corresponding to TGA curve analysis, this peak was mainly caused by the breakage of both non-covalent bonds and covalent bonds in QPI. QPI obtained the maximum decomposition rate at the DTG peak [23]. The decomposition rate of the control was the highest and the decomposition rate of QPI treated at 121 °C was the smallest, which was consistent with the results of TGA. Similar results were reported by Zhang et al., (2019) [47]. The possible reasons for this may be due to hydrothermal treatment of QPI led to protein defolding and subsequent cross-linking of denatured protein molecules, resulting in higher thermal stability [21]. In conclusion, hydrothermal treatment increased the thermal stability of QPI. In addition, Mir et al., (2021) also found that the thermal stability of QPI was significantly improved after heat treatment by a DSC study of quinoa protein [25]. This is consistent with our results in this study.

#### 3.1.3. Intrinsic Fluorescence Spectrum

**Figure 3:**
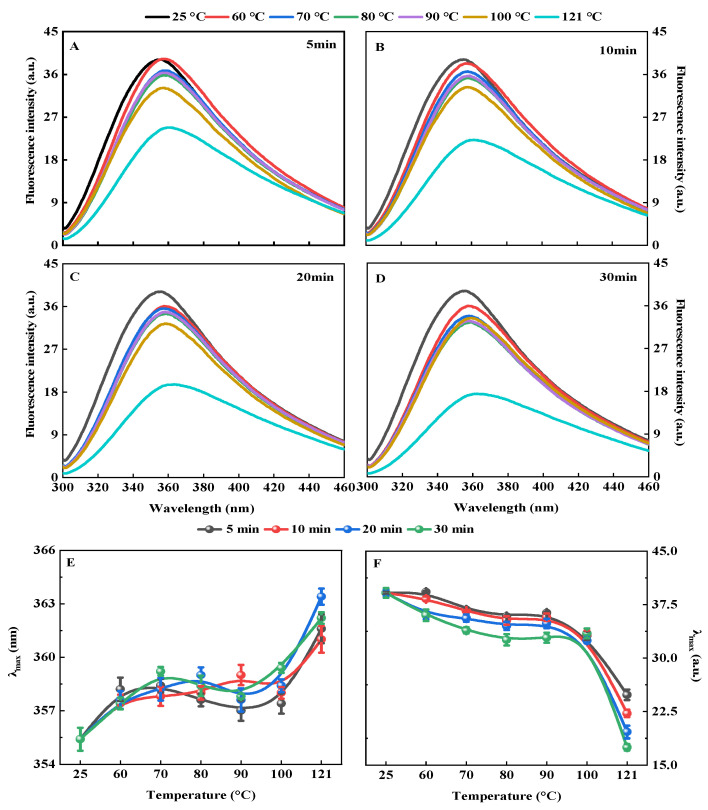
Effects of hydrothermal treatment of 5 min (**A**), 10 min (**B**), 20 min (**C**), and 30 min (**D**) on endogenous fluorescence spectrum, maximum absorption wavelength (**E**), and maximum fluorescence intensity (**F**) of QPI.

The QPI after hydrothermal treatment had the same peak shape as the control (Figure 3A–D). However, the hydrothermal treatment significantly affected its maximum absorption wavelength and its corresponding maximum fluorescence intensity. The maximum absorption wavelength of QPI increased gradually with the increase in hydrothermal treatment time and temperature, and reached the maximum when the hydrothermal treatment condition was 121 °C for 20 min, which was 2.25% higher than that of the control (Figure 3E). The maximum fluorescence intensity of QPI decreased gradually with the increase of hydrothermal treatment time and temperature, and dropped to the lowest when the hydrothermal treatment condition was 121 °C for 30 min, which was lower than that of control by 55.3% (Figure 3F). This was probably because hydrothermal treatment increased the hydrophobicity of the QPI and subsequently enhanced the intermolecular hydrophobic interactions of the exposed tryptophan residues, resulting in a decrease in intrinsic fluorescence intensity [21]. A similar phenomenon was observed in the study of heat treatment on sunflower protein isolates near an isoelectric point [46]. However, the results are in contradiction with the results of Chao et al., (2018) who observed a reverse kind of trend whereby the 100 °C pretreated cowpea protein isolates had an increased fluorescence intensity at pH 7.0 when compared to the untreated protein [48]. A plausible reason is that the QPI used in this work had higher surface hydrophobicity, which could enhance protein–protein interactions as compared to the cowpea proteins.

In addition, this study found that hydrothermal treatment at 121 °C for 20 min had the most significant effect on the endogenous fluorescence intensity of tryptophan in QPI, and the maximum endogenous fluorescence emission wavelength was significantly red-shifted (increased from 355.4 nm to 363.4 nm), which was 2.25% higher than that of the control. This result indicated that the degree of denaturation of QPI was greater at this time, the tertiary structure of QPI was destroyed, and tryptophan residues were gradually exposed on the protein surface. At the same time, some amide groups initially located on the main peptide chain of the protein were exposed [49]. A similar phenomenon was observed in the study of heat treatment on the tertiary structure of salt-soluble proteins of Pacific oyster (*Crassostrea gigas*) [50]. This suggests that hydrothermal treatment could alter the tertiary structure of QPI.

#### 3.1.4. Ultra-Violet (UV) Spectrum

**Figure 4:**
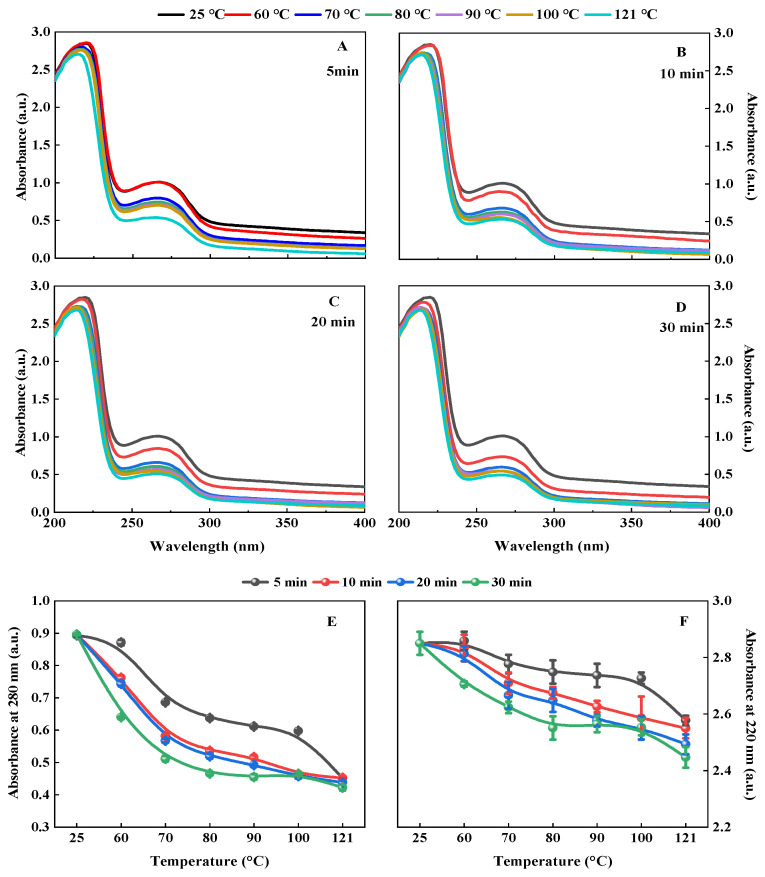
Effects of hydrothermal treatment of 5 min (**A**), 10 min (**B**), 20 min (**C**), and 30 min (**D**) on the UV absorption spectrum of QPI and the absorption value at wavelengths of 280 nm (**E**) and 220 nm (**F**), respectively.

In this study, we found that the hydrothermally treated QPI had the same UV absorption peak shape as the control, but its absorption peak intensity decreased with the increase in hydrothermal treatment temperature and time (Figure 4A–D). This is probably because the tyrosine or tryptophan content of QPI decreases during the hydrothermal treatment [51]. The minimum absorption peaks at 220 nm and 280 nm were observed in QPI hydrothermally treated at 121 °C for 30 min, which were 5.07% and 6.35% lower than the control, respectively (*p* < 0.05) (Figure 4E,F). Furthermore, the wavelength of the maximum absorption peak near 220 nm was blueshifted by 6 nm compared to the control. This is probably due to the aggregation of the microstructure of QPI by high-temperature treatment, where the color-emitting groups are wrapped and the UV-absorbing groups are reduced [52]. It was shown that hydrothermal treatment reduced the tyrosine, phenylalanine and tryptophan in QPI and the framework structure of QPI was changed. However, the result was inconsistent with the report of He et al., (2014). They observed that the UV absorption peak intensity of rapeseed protein increased after heat treatment [53]. In addition, when compared to the native rapeseed protein, the near-UV CD spectra peak at 262 nm underwent a red shift of 3–5 nm after heat treatment. The possible reasons for this may be due to the difference in the type and quantity of amino acids contained in rapeseed protein and QPI.

#### 3.1.5. Fourier Transform Infrared Spectrum (FTIR)

**Figure 5:**
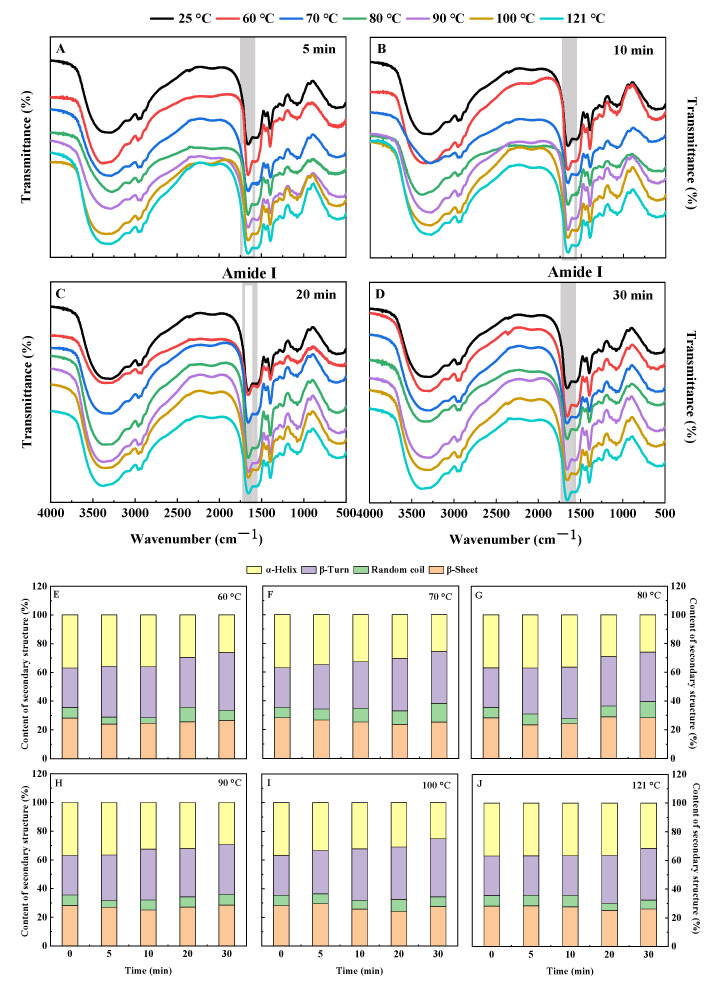
Effects of hydrothermal treatment of 5 min (**A**), 10 min (**B**), 20 min (**C**), and 30 min (**D**) on the Fourier infrared spectrum of QPI, 60 °C (**E**), 70 °C (**F**), 80 °C (**G**), 90 °C (**H**), 100 °C (**I**) and 121 °C (**J**) on the relative content of the secondary structure of QPI.

Conformational information on protein secondary structure could be efficiently analyzed by FTIR [54]. Figure 5A–D showed the original infrared spectra of QPI for different hydrothermal treatment conditions. Previous studies have shown that the FTIR region of the amide I band corresponds to the secondary structure in proteins as follows: 1610–1640 cm^−1^ belongs to β-sheet, 1660–1670 cm^−1^ belongs to β-turn, 1650–1658 cm^−1^ belongs to α-helix, and 1640–1650 cm^−1^ belongs to random coil [31]. The deconvolution and curve-fitting of the amide I region of QPI to obtain its second derivative spectrum (Figure S1). The relative content of each secondary structure was obtained according to the second derivative spectrum of the amide I band of QPI (Figure 5E–H).

The relative contents of α-helix, β-sheet, β-turn, and random coil in the secondary structure of QPI all changed significantly after hydrothermal treatment (*p* < 0.05). When the temperature was kept constant, the relative contents of α-helix in the QPI secondary structure gradually decreased with the increase in heat treatment time, while the relative contents of β-turn showed the opposite trend. In addition, the changing trends of the relative contents of β-sheets and random coils were irregular with the increase of heat treatment time. However, the relative contents of β-sheet in the QPI after hydrothermal treatment were lower than the control. In addition, when the temperature was lower than 100 °C and the hydrothermal treatment was performed for 30 min, the relative contents of random coils of QPI were greater than that of the control (Figure 5E–H). This is probably due to the destruction of the hydrogen bonds between adjacent peptide chains in the QPI during the heating process, resulting in the unfolding of the most compact α-helix in the protein molecule and the β-sheet aggregated inside the protein [55]. The relative content of α-helix in the protein gradually decreased with the heating time, which is consistent with our results. Moreover, similar results were found in the study of the effect of heat treatment on the secondary structure of camelina seeds protein isolates [56].

The results of this study indicated that the α-helix and β-sheet of QPI were transformed into β-turn and random coil. This structural change might be related to the denaturation of molecules in QPI under hydrothermal treatment [46]. The protein molecules that are denatured have their internal hydrogen bonds broken, and the protein molecules are unfolded, while the α-helix and β-sheet structures mainly use hydrogen bonds as the force, so the breakdown of hydrogen bonds leads to a decrease in the content of both [57]. Furthermore, β-turn and random coil may be transformed from more ordered structural units, and the β-sheet between the molecules of the thermal aggregates is also easily transformed into β-turn, which leads to an increase in the relative content of β-turn and random coil [58]. It is inferred that β-turn and random coil play an important role in the formation of thermal aggregates. Moreover, the findings of this study are consistent with the results of Mir et al., (2021) [25]. In the study of the secondary structure changes of

QPI after heat treatment by circular dichroism, they found that the secondary structure of native and heat–treated QPI was dominated by α-helix and β-sheet, and heat treatment led to the destruction of α-helix in the secondary structure of QPI.

### 3.2. Determination of Functional Properties of QPI

#### 3.2.1. Solubility

Solubility, as the basis for other functional properties of proteins, is one of the most important functional properties of proteins and has a very close relationship with emulsification, foaming, and other properties of proteins. It also accurately reflects the degree of aggregation of proteins and whether their internal structure is denatured [27]. It is generally believed that proteins aggregate after heat treatment, thereby reducing solubility. However, some studies have shown that moderate heat treatment could improve the solubility of proteins [59]. In this study, we found that when the heat treatment time was kept constant, the solubility of QPI showed a trend of first increasing and then decreasing in the range of 60–121 °C, and reached a maximum of 90 °C for 30 min, which was higher than that of the control by 33.4% (*p* < 0.05). Previous studies showed that the solubility of soybean proteins after treatment at 85 °C was higher than those of samples treated at 55 °C [60]. This is probably due to the fact that poorly water-soluble proteins expose more hydrophilic groups after proper heat treatment, and these groups can subsequently interact with water, leading to a higher water solubility [61].

**Figure 6:**
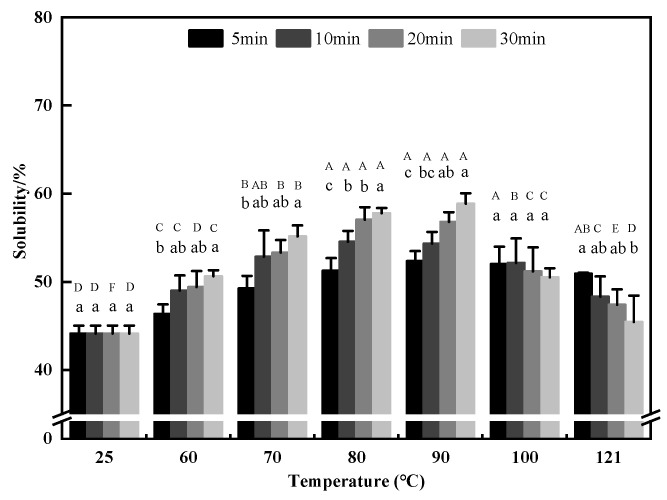
Effects of different hydrothermal treatment conditions on the solubility. A–F indicates the significant differences between different temperatures and a–c indicates the significant differences between different times (*p* < 0.05).

When the temperature was 100 °C and 121 °C, the solubility of QPI decreased slowly with the increase in hydrothermal treatment time, and finally reduced to the minimum at 121 °C for 30 min (Figure 6). Similar results were reported by Lv et al., (2017) [62]. The possible reason for this may be that the high temperature causes the protein to form a large number of insoluble aggregates. This is consistent with the findings of Yu et al. (2021) on the effect of heat treatment on the solubility of soybean protein. This suggests that high–temperature treatment can reduce protein solubility [63]. In addition, the results indicated that the solubility of QPI extracted by alkali-soluble acid precipitation ranged from 28.34% to 78.46%, while the solubility of the native QPI was 44.12%. This may be due to differences in extraction pH, which lead to changes in the interaction between the protein and water, resulting in different solubility [6].

#### 3.2.2. Water-Holding Capacity (WHC) and Oil-Holding Capacity (OHC)

WHC and OHC are the ability of a substance to bind water and oil under limited water and oil conditions [64]. In this study, it was found that the WHC and OHC of QPI after hydrothermal treatment were significantly higher than those of the control, and the WHC and OHC of QPI increased first and then decreased with the increase in temperature. In the range of 60–90 °C, the WHC and OHC of QPI increased gradually with the increase in hydrothermal treatment time and reached a maximum of 90 °C and 30 min, which were 12.50% and 14.18% higher than those of the control, respectively (*p* < 0.05) (Table 2). Previous studies found that the WHC and OHC of peanut seed albumin increased gradually with the increase in heat treatment temperature from 15 to 55 °C [65]. In addition, different heat treatments significantly increased the WHC of guar proteins [66]. This is probably due to the fact that the spatial structure of the protein is opened after heating, which allows some polar groups inside to be transferred to the surface, therefore increasing its WHC and OHC [67]. Furthermore, the findings of this study are consistent with the results of Cerdan et al., (2019) who observed that the WHC and OHC of the heat-treated QPI were 2-fold and 10-fold higher than those of the native QPI, respectively [24]. However, such an increase is much higher than the results of this study, which may be because they used a method of vacuum drying at 35 °C is different from the method in this study.

The WHC and OHC of QPI decreased gradually with the increase in hydrothermal treatment time when the temperature was higher than 90 °C, and the minimum values of 145% and 157% were reached when the heat treatment conditions were 121 °C for 30 min, respectively. It was also found that excessive temperature could significantly reduce its WHC and OHC in the study of heat–treated sunflower protein [20]. This is probably due to the complete denaturation of the protein at high temperature, leading to the exposure of the hydrophobic groups hidden inside, which leads to the reduction in WHC and OHC. Moreover, the OHC of the QPI in this study was slightly higher than that in the previous report [29], which is probably due to the different quinoa varieties used, and the protein content and composition were different. In this study, the trends of WHC and OHC of QPI were found to be in good agreement with solubility. Therefore, we speculate that the WHC and OHC of QPI might be related to its solubility.

#### 3.2.3. Emulsifying Activity (EA) and Emulsion Stability (ES)

EA and ES characterize the ability of protein to adsorb to the oil-water interface and to form a stable emulsion, respectively [68]. In this study, we found that the EA and ES of QPI after hydrothermal treatment were significantly higher than those of the control. When the temperature was less than 90 °C, the EA and ES of QPI increased with the increase in temperature, and reached a maximum of 90 °C for 30 min of hydrothermal treatment, which was significantly higher than those of the control by 84.4% and 27.1% (*p* < 0.05) (Table 3 and Table 4). This is similar to the results of the study in which the ES of faba bean protein concentrate heat-treated at 95 °C for 15 min was significantly higher than that of the control [26]. However, when the temperature was 100 °C and 121 °C, the EA and ES of QPI decreased gradually with the extension of hydrothermal treatment time, and the minimum value was reached at 121 °C, 30 min heat treatment; at which time, the EA was lower than the control by 5.95%, while the ES was higher than the control by 10.1% (*p* < 0.05). The reason for this result might be a change in the solubility of QPI [69].

It was shown that the EA and ES of vicilin-rich proteins isolated from kidney beans after high-temperature treatment were elevated under moderate heating conditions and decreased after excessive heating [70]. The possible reasons for this may be because the moderate heating induced structural changes in the protein in favor of EA and ES, which might be the driving force for improving the EA and ES of the protein [29]. A hydrothermal treatment temperature greater than 90 °C will cause more unfolding of the protein, exposing their internal hydrophobic groups, resulting in a decrease in solubility, which in turn reduces EA and ES [71]. Therefore, we speculate that protein solubility might have an effect on its EA and ES. The above results show that the EA and ES of QPI could be improved by heat treatment below 121 °C, which is similar to the results of Mir et al., (2021) [25]. They found that the EA and ES of QPI increased significantly after water a bath treatment at 80, 90 and 100 °C for 15 and 30 min

## 4. Conclusions

In this study, the QPI after hydrothermal treatment was studied from the perspective of structure and functional properties. The results indicated that hydrothermal treatment had significant effects on both the structural and functional properties of QPI. Hydrothermal treatment at 60–121 °C for 5–30 min increased the particle size and thermal stability of QPI, and significantly changed its secondary and tertiary structures. In addition, hydrothermal treatment at 60–90 °C for 5–30 min had a positive effect on improving the functional properties of QPI such as WHC, OHC, EA, ES, and solubility, while hydrothermal treatments at 100 and 121 °C damaged these properties of QPI. Overall, the functional properties of the QPI after hydrothermal treatment were all superior to those of the control. Moreover, several other functional properties of QPI appeared to depend on the the its solubility. Insights gained from this study may help improve the functional properties of QPI by adjusting the hydrothermal treatment conditions. Moreover, our findings provide further support for studying the structure and functional properties of QPI after hydrothermal treatment, which is crucial for their application in food. These findings demonstrate that QPI could be added to foods involving thermal processing. Furthermore, based on the abundant nutritional value of QPI and its good functional properties after heat treatment, it can also be added to functional foods to increase the added value of the product.

## Figures and Tables

**Table 1:** Effects of different hydrothermal treatment conditions on D_[4,3]_ and D_[5,0]_, nm. A–F indicates the significant differences between different temperatures and a–c indicates the significant differences between different times (*p* < 0.05).

Size (nm)	Temperature (°C)	Time (min)			
		5	10	20	30
	25	11.54 ± 1.10 ^Ea^	11.54 ± 1.10 ^Fa^	11.54 ± 1.10 ^Ea^	11.54 ± 1.10 ^Fa^
	60	13.57 ± 0.53 ^Db^	14.23 ± 0.99 ^Eab^	14.35 ± 0.59 ^Dab^	15.67 ± 1.09 ^Ea^
	70	14.78 ± 0.65 ^Db^	16.13 ± 0.53 ^Db^	16.44 ± 1.10 ^Cb^	18.61 ± 0.96 ^Da^
D_[4,3]_	80	16.76 ± 0.64 ^Cc^	18.84 ± 0.98 ^Cb^	21.09 ± 0.79 ^Ba^	22.01 ± 0.77 ^Ca^
	90	18.11 ± 0.45 ^Cb^	20.59 ± 1.11 ^BCb^	21.24 ± 0.61 ^Bb^	21.64 ± 1.30 ^Ca^
	100	20.20 ± 0.31 ^Bc^	21.87 ± 0.48 ^Bb^	20.52 ± 0.46 ^Bc^	24.47 ± 0.74 ^Ba^
	121	34.49 ± 0.41 ^Ac^	143.02 ± 0.49 ^Ab^	49.58 ± 0.41 ^Aa^	49.69 ± 0.87 ^Aa^
	25	7.37 ± 0.52 ^Da^	7.37 ± 0.52 ^Da^	7.37 ± 0.52 ^Da^	7.37 ± 0.52 ^Ea^
	60	10.90 ± 0.40 ^Cb^	9.62 ± 0.54 ^Cc^	10.04 ± 0.43 ^Cbc^	12.94 ± 0.62 ^Da^
	70	9.69 ± 0.44 ^Cc^	10.37 ± 0.45 ^Cbc^	10.92 ± 0.34 ^Cb^	11.95 ± 0.62 ^Da^
D_[5,0]_	80	9.85 ± 0.36 ^Cb^	10.36 ± 0.46 ^Cb^	14.60 ± 0.30 ^Ba^	15.49 ± 0.35 ^Ca^
	90	9.84 ± 0.13 ^BCd^	13.05 ± 0.44 ^Bc^	14.87 ± 0.37 ^Bb^	15.72 ± 0.35 ^Ca^
	100	12.33 ± 0.28 ^Bd^	13.94 ± 0.36 ^Bc^	15.03 ± 0.18 ^Bb^	17.87 ± 0.61 ^Ba^
	121	24.51 ± 0.42 ^Ac^	30.55 ± 0.39 ^Ab^	36.16 ± 0.16 ^Aa^	36.36 ± 0.65 ^Aa^

**Table 2:** Effects of different hydrothermal treatment conditions on the water–holding capacity and oil–holding capacity, %. A–E indicates the significant differences between different temperatures and a–c indicates the significant differences between different times (*p* < 0.05).

Capacity (%)	Temperature (°C)	Time (min)			
		5	10	20	30
	25	143.53 ± 2.30 ^Da^	143.53 ± 2.30 ^Aa^	143.53 ± 2.30 ^Ea^	143.53 ± 2.30 ^Ea^
	60	144.80 ± 0.95 ^Da^	145.33 ± 3.97 ^Aa^	145.87 ± 1.52 ^Da^	146.07 ± 1.08 ^Da^
	70	147.40 ± 0.31 ^Cb^	152.47 ± 2.36 ^Aa^	152.07 ± 0.72 ^Ca^	152.80 ± 0.95 ^Ca^
water	80	153.73 ± 1.54 ^Ba^	153.93 ± 3.62 ^Aa^	154.27 ± 0.74 ^Ba^	157.20 ± 2.60 ^Ba^
	90	153.00 ± 3.85 ^Bb^	158.13 ± 1.83 ^Aa^	161.67 ± 0.85 ^Aa^	161.47 ± 1.15 ^Aa^
	100	161.47 ± 3.62 ^Aa^	152.13 ± 0.99 ^Ab^	147.60 ± 1.00 ^Dc^	145.73 ± 1.14 ^Dc^
	121	152.33 ± 5.28 ^Ba^	151.20 ± 0.47 ^Aab^	146.53 ± 0.92 ^Dab^	145.00 ± 3.59 ^DEc^
	25	151.80 ± 0.64 ^Ea^	151.80 ± 0.64 ^Ea^	151.80 ± 0.64 ^Ea^	151.80 ± 0.64 ^Ea^
	60	152.87 ± 2.16 ^DEa^	154.33 ± 1.90 ^Da^	155.40 ± 3.60 ^Da^	156.00 ± 1.40 ^Da^
	70	154.00 ± 1.87 ^Db^	154.53 ± 0.63 ^Db^	155.80 ± 2.17 ^Dab^	159.87 ± 3.67 ^Ca^
oil	80	159.27 ± 2.16 ^Cb^	166.73 ± 3.38 ^Aa^	167.40 ± 2.06 ^Aa^	168.00 ± 1.56 ^Ba^
	90	163.40 ± 0.36 ^Bc^	166.20 ± 1.17 ^Abc^	167.67 ± 2.33 ^Ab^	173.33 ± 2.42 ^Aa^
	100	171.33 ± 2.73 ^Aa^	162.20 ± 5.46 ^Bb^	162.13 ± 4.30 ^Bb^	157.07 ± 0.90 ^Db^
	121	160.60 ± 2.54 ^Ca^	159.07 ± 4.03 ^Ca^	159.67 ± 5.49 ^Ca^	156.60 ± 1.56 ^Da^

**Table 3:** Effects of different hydrothermal treatment conditions on the emulsifying activity, m^2^·g^−1^. A–D indicates the significant differences between different temperatures and a–c indicates the significant differences between different times (*p* < 0.05).

Temperature (°C)	Time (min)			
	5	10	20	30
25	6.55 ± 0.38 ^Da^	6.55 ± 0.38 ^Da^	6.55 ± 0.38 ^Ca^	6.55 ± 0.38 ^Da^
60	7.65 ± 0.25 ^CDb^	7.72 ± 0.39 ^CDb^	8.06 ± 0.73 ^BCb^	9.17 ± 0.30 ^BCa^
70	7.95 ± 0.28 ^BCDc^	8.73 ± 0.46 ^BCbc^	9.46 ± 0.52 ^Bb^	10.57 ± 0.64 ^ABa^
80	9.56 ± 0.68 ^ABb^	10.19 ± 0.40 ^ABb^	11.57 ± 0.71 ^Aa^	11.68 ± 0.66 ^Aa^
90	11.05 ± 0.38 ^Aa^	11.76 ± 0.67 ^Aa^	11.82 ± 0.58 ^Aa^	12.05 ± 0.38 ^Aa^
100	10.31 ± 0.59 ^Aa^	9.84 ± 0.80 ^Bab^	8.68 ± 0.36 ^Bb^	8.64 ± 0.59 ^Cb^
121	9.28 ± 0.29 ^ABCa^	8.46 ± 0.37 ^BCab^	7.72 ± 0.39 ^BCb^	6.16 ± 0.56 ^Dc^

**Table 4:** Effects of different hydrothermal treatment conditions on the emulsion stability, %. A–F indicates the significant differences between different temperatures and a–c indicates the significant differences between different times (*p* < 0.05).

Temperature (°C)	Time (min)			
	5	10	20	30
25	56.08 ± 0.59 ^Da^	56.08 ± 0.59 ^Da^	56.08 ± 0.59 ^Ca^	56.08 ± 0.59 ^Fa^
60	57.13 ± 0.88 ^CDb^	64.37 ± 2.70 ^Ca^	65.87 ± 0.68 ^Ba^	66.10 ± 2.14 ^Ca^
70	58.13 ± 1.73 ^Cb^	65.34 ± 2.30 ^BCa^	68.51 ± 0.86 ^Aa^	68.00 ± 0.41 ^Ba^
80	63.57 ± 0.66 ^Bb^	68.62 ± 1.42 ^Aa^	68.86 ± 1.06 ^Aa^	64.21 ± 0.50 ^Db^
90	64.15 ± 1.10 ^Bb^	69.63 ± 0.39 ^Aab^	68.62 ± 1.42 ^Aab^	71.25 ± 1.60 ^Aa^
100	70.49 ± 1.74 ^Aa^	69.27 ± 1.45 ^Aa^	67.37 ± 0.74 ^ABab^	64.48 ± 1.79 ^CDb^
121	70.55 ± 0.45 ^Aa^	66.31 ± 2.20 ^Bb^	65.80 ± 2.09 ^Bb^	61.76 ± 1.27 ^Ec^

## Data Availability

The data presented in this study are available on request from the corresponding author.

## Supplementary Materials

The following supporting information can be downloaded at: https://www.mdpi.com/article/10.3390/foods11192954/s1, Figure S1: Deconvolution and curve fitting of the Amide I region for QPI with different hydrothermal treatment conditions.
