# Supplementary material for: Effect of Hydrothermal Treatment on the Structure and Functional Properties of Quinoa Protein Isolate
Source: Foods. 2022 Sep 21;11(19):2954. doi: 10.3390/foods11192954 (PMC9563563; doi:10.3390/foods11192954)
Supplement: Supplementary file 1 [file foods-11-02954-s001.zip › foods-1912584-supplementary.pdf]

Supplementary Materials

Figure Captions

Figure S1 Deconvolution and curve-fitting of the Amide I region for QPI with different hydrothermal treatment conditions. (Curves with different colors are automatically generated spectra after PEAK FIT fitting, and have no actual representative meaning.)

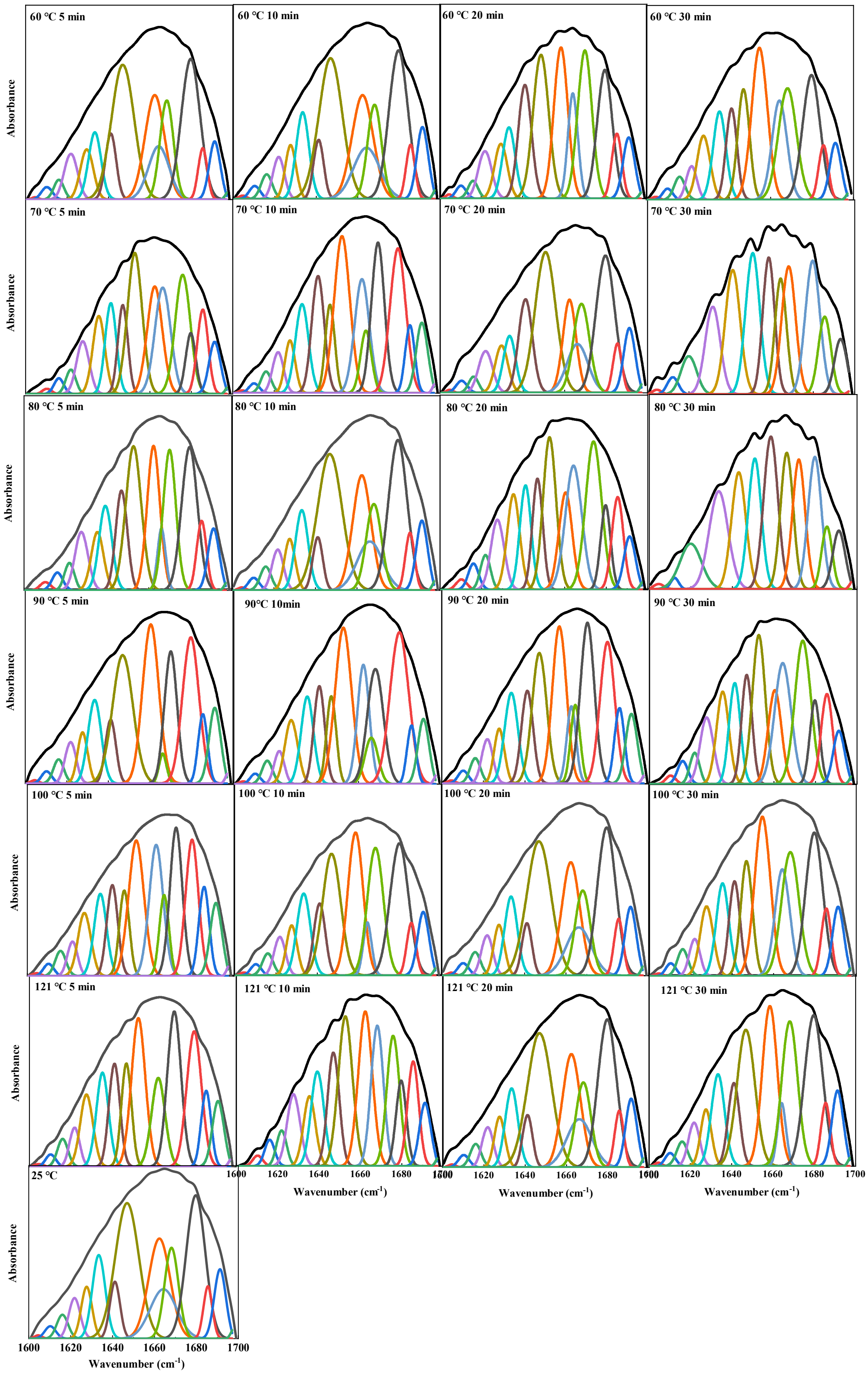
60 °C 5 min
60 °C 10 min
60 °C 20 min
60 °C 30 min
70 °C 5 min
70 °C 10 min
70 °C 20 min
70 °C 30 min
80 °C 5 min
80 °C 10 min
80 °C 20 min
80 °C 30 min
90 °C 5 min
90°C 10min
90 °C 20 min
90 °C 30 min
100 °C 5 min
100 °C 10 min
100 °C 20 min
100 °C 30 min
121 °C 5 min
121 °C 10 min
121 °C 20 min
121 °C 30 min
25 °C
Absorbance
1600
1620
1640
1660
1680
1700
Wavenumber (cm-1)

Figure S1.
